# Digital storytelling: a tool for health promotion and cancer awareness in rural Alaskan communities

**DOI:** 10.3402/ijch.v74.28781

**Published:** 2015-09-04

**Authors:** Melany Cueva, Regina Kuhnley, Laura Revels, Nancy E. Schoenberg, Mark Dignan

**Affiliations:** 1 Community Health Aide Program, Alaska Native Tribal Health Consortium, Anchorage, AK, USA; 2 Clinical & Research Services, Alaska Native Tribal Health Consortium, Anchorage, AK, USA; 3 Behavioral Science, University of Kentucky, Lexington, KY, USA; 4 Department of Internal Medicine, College of Medicine, University of Kentucky, Lexington, KY, USA

**Keywords:** digital storytelling, storytelling, cancer communication, community cancer education, Alaska Native, indigenous research, cancer education materials, digital storytelling as adult education, digital storytelling as health promotion, cancer-related digital stories

## Abstract

**Background:**

The purpose of this study was to learn community members’ perspectives about digital storytelling after viewing a digital story created by a Community Health Aide/Practitioner (CHA/P).

**Methods:**

Using a qualitative research design, we explored digital storytelling likeability as a health-messaging tool, health information viewers reported learning and, if viewing, cancer-related digital stories facilitated increased comfort in talking about cancer. In addition, we enquired if the digital stories affected how viewers felt about cancer, as well as if viewing the digital stories resulted in health behaviour change or intent to change health behaviour.

**Findings:**

A total of 15 adult community members participated in a 30–45 minute interview, 1–5 months post-viewing of a CHA/P digital story. The majority (13) of viewers interviewed were female, all were Alaska Native and they ranged in age from 25 to 54 years with the average age being 40 years. Due to the small size of communities, which ranged in population from 160 to 2,639 people, all viewers knew the story creator or knew of the story creator. Viewers reported digital stories as an acceptable, emotionally engaging way to increase their cancer awareness and begin conversations. These conversations often served as a springboard for reflection, insight, and cancer-prevention and risk-reduction activities.

Digital storytelling, which has gained momentum as an education (1, 2) and health-promotion tool (3, 4), is an emerging pathway for Alaska Community Health Aides and Community Health Practitioners (CHA/Ps) to share culturally respectful health messages with people in their rural communities. As an innovative method for community health advocacy, Alaska Native people's dynamic storytelling traditions were combined with cancer education for CHA/Ps, merging storytelling customs with computer technology as a powerful new way to share cancer knowledge within Alaska's communities. Digital storytelling can preserve and promote indigenous wisdom, celebrating myriad stories, lived experiences, and life worlds while engaging community members and developing capacities for health behaviour change (5, 6). In these ways, the locus of control is centred within the hearts and minds of the story creators, and situated within the lived context of her or his community. Linking research and practice, as an innovative approach to address health disparities, digital storytelling respects indigenous knowledge, voices and experiences to locate power within the indigenous community (7, 8). Each participant of the cancer education and digital storytelling course selects personally chosen images, photos and music to create a 2–3 minute authentic, vivid and culturally relevant audio-visual documentary that gives voice to their lived stories not only at present but may also become part of cultural history, tradition and wisdom in coming generations.

The purpose of this study was to learn community members’ perspectives about digital storytelling after viewing community health care providers’ digital stories. Using a qualitative research design (9), we explored digital storytelling likeability as a health-messaging tool, health information viewers reported learning and, if viewing, cancer-related digital stories facilitated increased comfort in talking about cancer. In addition, we enquired if the digital stories affected how viewers felt about cancer, as well as if viewing the digital stories resulted in their own health behaviour change or intent to change health behaviour.

## Cancer-related digital stories created by Alaska's CHA/Ps

Over half of Alaska Native people live in rural communities separated by vast distances. The majority of these 178 communities are not located on a road system and are only accessible year-round primarily by air transportation. To meet community members’ basic health care needs, Alaska has a unique, longstanding community-based system to deliver local health care to Alaska Native communities throughout the state. CHA/Ps are selected by their communities and employed as primary providers of emergency, basic, acute, preventive and chronic disease health care (10). To become a CHA/P, community members, selected by their tribes, participate in an intensive 15-week basic medical training curriculum which is divided into four 3–4 week sessions. Completion of the 4 sessions and practicum takes approximately 2 years. Since the 600 hours of CHA/P basic medical training include only 2 hours of cancer education, CHA/Ps have expressed an on-going and urgent need for cancer education. CHA/Ps participate in continuing education to stay abreast of current medical practice and recommendations for screening and health maintenance. As members of their communities, CHA/Ps are ideally situated not only to provide medical care within the social and cultural context of their communities but also to advocate, educate and promote health and wellness throughout the community. As one CHA/P noted: “A lot of communities look to the Health Aide as educators and mentors for healthy lifestyles.”

Cancer, considered a rare disease among Alaska Native people in the 1950s (11), surpassed heart disease in the 1990s to become the leading cause of death among Alaska Native people, all ages combined (12). Data from 2004 to 2008 showed that Alaska Native cancer mortality rates were 32% higher than those for US Whites. In addition, age-adjusted cancer incidence rates between 1974 and 2008 for Alaska Native people have increased for all sites by 33% (133% for breast, 83% for prostate, 67% for lung and 13% for colorectal). Throughout this period (1974–2008), Alaska Native incidence rates for lung and colorectal cancer have been higher than US White rates. In the 5 years period of 2004–2008, lung cancer rates were 94.8 for Alaska Native people versus 57.8 for US Whites, while colorectal cancer rates were 87.9 for Alaska Native people versus 45.1 for US Whites. Alaska Native breast cancer incidence rates, which were lower historically, are now similar to US White rates during the 2004–2008 period (67.6 for Alaska Native people vs. 68.3 for US Whites) (12).

## Theoretical framework

The “narrative as culture-centric health-promotion model” (13) provided a theoretical basis for the development and sharing of CHA/P digital stories. Honouring cultural knowledge is at the core of the narrative model and provides a strong foundation for creating and sharing life lessons and experiences. CHA/P digital story creation is culture-centric, defined within the model as messages that reflect a within-culture view, drawing directly from personal stories and experiences. Narrative characteristics identified as engaging elements within the model are defined as realism, likeability, homophily (like self), generation of empathy and cultural appropriateness. In addition, not emphasized within the model but at the heart of indigenous epistemology (how we think about or know reality) and ontology (the way that we view reality) is the importance of relationships and interconnections (14) which is reflected in how digital stories are told and shared.

## Methods

This research protocol was reviewed and approved by the Alaska Area Institutional Review Board and the South Central Foundation (SCF) Executive Committee and the SCF Board of Directors. In addition, this manuscript was reviewed and approved by the Alaska Native Tribal Health Consortium (ANTHC) Health Research Review Committee (HRRC) on behalf of the ANTHC Board of Directors and the SCF Executive Committee and the SCF Board of Directors.

### Cancer education and digital storytelling courses

To support CHA/Ps and community members in addressing the problem of cancer in Alaska Native communities, four 5-day cancer education courses incorporating digital storytelling as a health-promotion tool were offered between March and October 2014. This face-to-face course was held in 3 locations (Anchorage, Nome and Bethel) and was attended by a total of 30 CHA/Ps from diverse communities located throughout Alaska. Each course participant created a heartfelt digital story using free computer software. CHA/P course participants recorded, in their own voices, a 200–250 word scripted story they had written and then added their own photos and music to create a short 2–3 minute movie. Topics of digital stories included wellness ways, cancer risk reduction and prevention, and screening for early detection and treatment. All participants, with the help of the 2 course facilitators, regardless of computer skills prior to the course, were successful in creating a digital story, which they shared post-course with people in their communities. At the conclusion of the digital storytelling course, participants were asked to invite community members who had viewed their stories to participate in a telephone interview with a project team member. With community member permission, 10 course participants provided viewer contact information for the team member to conduct an interview. The use of the word “viewer” within this manuscript refers only to viewers of digital stories who were interviewed for this study. Timing of viewer interviews depended upon when course participants provided viewer information after the course and upon the availability of viewers for an interview. Viewer interviews were conducted by telephone between 1 and 5 months after the CHA/P completed the cancer education and digital storytelling course.

### Interview script

To learn community viewers’ perspectives about digital storytelling as a health-promotion and cancer awareness tool, a semi-structured interview guide was developed by the project team with input from 5 of the 6 people (1 person was lost to follow-up) who had previously participated in a CHA/P cancer education and digital storytelling course provided in Anchorage March 2013, prior to this study. With viewer permission, contact information was provided by 2 course participants for the project team to contact 2 digital story viewers. The 2 adult community members who had viewed a digital story created as part of the March 2013 course provided comments about the interview script. CHA/Ps and viewers commented on the questions including wording and order which resulted in the following interview script (Table I).

**Table I:** Interview script

Thank you very much for sharing a little more with us about digital storytelling. Thinking back about the digital story that you watched …
• Is there anything in particular that stuck with you about the story?
• How do you know the story teller (friend, family, co-worker) …
• Where did you see the story (clinic, community setting, one-on-one) …
• How do you feel about digital storytelling as a way to learn more about cancer?
• Are digital stories a culturally respectful way to receive cancer health messages?
• Do you feel differently about cancer or cancer prevention after watching the digital story? Please share a little more about that …
• Do you think digital stories make it easier to talk about cancer? … Please share a little more about that …
• After watching the digital story, do you feel like you might do anything differently? Please share a little more about that …
• What else would you like us to know about digital storytelling?

### Viewer recruitment

Upon completion of the cancer education and digital storytelling course, CHA/Ps were asked to identify adult community members who had watched their digital story and would be willing to be interviewed by a member of the project team. The CHA/P, with viewer permission, provided the project team with the name and contact information of the community viewer. CHA/Ps could choose how and when they wanted to show their digital story to best support community outreach.

### Viewer interviews

All interviews were conducted by a nurse practitioner who is also a Community Health Aide Instructor with over 20 years of experience in conducting qualitative interviews with Alaska Native people. The interviewer was not part of the cancer education and digital storytelling course implementation.

Interviews were conducted in English. With viewer consent, all interviews were audio-recorded and transcribed. Viewers verbally agreed for the project team to anonymously share their ideas with a wider audience interested in learning more about digital storytelling. After each interview was completed, a thank you card and a VISA gift card were sent acknowledging the project team's appreciation for the viewers’ time and contribution in helping us to learn more about digital storytelling.

The project team, which included co-instructors of the cancer education and digital storytelling course, the interviewer and an experienced qualitative researcher, reviewed viewer transcriptions or summaries, first independently and then collectively, to identify emergent themes. Thematic analysis was used to ground the analysis within the context of participants’ stated experiences. Following this initial approach to coding, a more focused content analysis was incorporated using Larkey's narrative model (13) and indigenous theory that emphasizes the importance of relationships (14). Interviews were conducted until it was agreed among the project team and the external project qualitative researcher that data saturation had been achieved.

## Findings

A total of 15 adult community members participated in a 30–45 minute interview. Viewer interviews were conducted from 1 to 5 months after the CHA/P completed the cancer education and digital storytelling course: 1 month post-course (2), 6 weeks post-course (4), 3 months post-course (4), 4 months post-course (2) and 5 months post-course (3). Most (13) of the viewers interviewed were female, all were Alaska Native, and they ranged in age from 25 to 54 years with an average age of 40 years. Due to the small size of communities, which ranged in population from 160 to 2,639 people, all viewers knew the story creator or knew of the story creator and self-identified as a friend (2), community member (3), co-worker (4) or family member (6). Viewers reported watching the digital story in a variety of settings including: Facebook (3), health clinic (6), website (1), home (1), community showing (2), local business (1) and work (1).

Described within this section are the emergent themes from viewer interviews. The 9-question script served as a conversation guide to understand digital storytelling likeability as a health-messaging tool, information learned and impact on cancer communication, attitudes and intent to change behaviour.

### Viewers’ perception of digital stories as culturally respectful and culturally relevant

Digital stories were well received by all viewers and described as being culturally respectful and culturally relevant because they were stories from people in their community or throughout Alaska. Viewers emphasized the importance of including Alaska Native cultural values – particularly storytelling traditions and respectful relationships within families and tribal communities. Viewers described digital stories as an effective way to share cancer understandings along with acknowledging family and community as a significant part of wellness.

I like how the digital stories relate to people in our region, local people telling their stories. It's good for the people to see the stories and know that we are from the community, and it's good for Alaska Native people to see the stories, our families and our communities and know what we're going through with cancer and that we can help.

The bond among people in Native culture is extremely important. People can really take it in when it is your family and close to home and you see the people you know and what happened.

I like the fact that all these stories are all of Alaska Natives and they all shared a story they've experienced within their family, and they want to bring this awareness of preventative kinds of care and get these cancer screenings done. By having cancer screenings we can keep ourselves healthy.

### Viewer's perspectives of digital storytelling as a way to learn about cancer

All viewers reported that watching digital stories was a good way to increase their awareness, knowledge and understanding of cancer. Specifically, the inclusion of personal stories and pictures from people's communities enhanced information retention.

I really loved the pictures, and the stories. It's more interesting than just reading statistics and helps keep the viewer's attention, allowing the information being provided to have a bigger impact … you can read words, but when you're watching, hearing the person's voice and the imaging, it really cements it in your head a lot better than print.

Viewers described detailed story content which had increased their knowledge and understanding of cancer. Through digital stories, viewers had internalized health messages with specific and relevant meaning to their lives, even relating details of the digital story to the project interviewer 5 months after they had watched a course participant's story.

The awareness is more in my mind now, because the digital story was explaining what the symptoms are and what to look for. I know now what to watch for, like symptoms and to get checked. But also get the check-up even before you could have symptoms – get the screenings.

I didn't know colon cancer ran in our family until I watched the story. It made me aware of getting checked.

### Viewer perceptions about cancer communication

All viewers related that talking about cancer was uncomfortable and challenging because of the lived experience with cancer disease and death within their close-knit communities. Digital stories, they reported, gave them a way to more openly and comfortably talk about cancer with others. Digital stories were reported as providing a pathway to begin difficult conversations or to understand diverse perspectives.

I have not been comfortable talking with people about cancer. My dad had pancreatic cancer and we just didn't talk about it. It's really scary. But after I saw the digital story, I felt like we need to talk about cancer more. I think this [digital story] is a good way to help ‘break the ice’ and get people more involved and better informed.

Personally I would feel more comfortable now to encourage people and talk with them and help them know that if you are diagnosed with something early on, then there are treatment options that can help with all the different types of cancers. There are treatments all along the way that will help you fight it.

I haven't known how to encourage people. But after I saw the digital story it just makes it easier to encourage people to get screened. I'm eager to get materials about cancer prevention and screening about cancer out to the people in the community now.

The stories are very effective, especially here where the word “cancer” instantly brings to mind, “Oh, it's death; cancer is death.” But there are people who survive with cancer and the stories show how it can be a positive thing that happens in people's lives.

The stories are good for helping people to know how to talk to providers, how to ask questions about treatment options. My mother would never ask questions. Please help our Tribal members learn that it is okay to ask questions and be involved in treatment options.

### Viewers’ feelings and perceptions of cancer after viewing digital stories

As viewers experienced a digital story, they often discovered a common thread within the narrative that was personally meaningful and affirming:

Inspiration

You can really take it in … and you see the people you know and what happened to them. So it's like everybody in the community can be inspired.

I had a close friend at the age of 27 who had breast cancer. When I was watching the movies it reminded me of my friend. She survived and is living and doing well, and she's learned from it.

The messages really inspired me. The stories just catch your heart and you want to take care of yourself.

Insight

Most feel that cancer happens to people outside of our family, but the stories remind us cancer is close to us. Cancer affects us all. Cancer can happen to anyone.

The empathy that happens in watching … you are drawn into the storyline and then hearing the words – it ties you to the story. Putting [cancer information] in digital storytelling changed my perspective. In watching the video it taught me to question more about how I feel and why I feel the way I do about cancer and death and modify how I feel and handle it.

Critical reflection

It was a sobering experience for me because I am a smoker and it seems to me that no one really expects anything like getting cancer to happen to them.

### Viewer health behaviours: changes made as a result of digital stories and intention to change health behaviours

As a result of watching a digital story, 6 viewers described wellness choices they had made or intended to make. In addition, all viewers reported encouraging and supporting family, friends and community members to consider and make healthy eating changes, quit/decrease smoking to reduce cancer risk and to have recommended screening exams for prevention and early detection of cancer. Viewers’ comments reflected cultural values of relationships among family and within community.

I quit smoking – I have an 18 year old daughter that I just found out is two months pregnant, and then, my 8 year old son talked to her and he made sure she quit smoking.

We have started teaching our kids to start having our fish, moose and caribou instead of all these processed foods. I've been trying to drink more water and less soda pop.

I think more about how the cigarette I'm smoking is harming my body and I am trying to cut down on the habit.

### Viewers’ comments on sharing digital stories to expanded communities of people

After watching digital stories, viewers offered ways to increase digital story exposure which included posting the stories on Facebook and showing the stories at health fairs and other community events. Viewers suggested linking the stories with additional information and resources. Digital stories piqued viewers’ interest, and they wanted to learn more information or to know how to schedule a recommended screening.

The thing about it is that it is really nice that you can watch it anywhere at any time with the technology we have now. It's a good way to reach out to the community. I think it's amazing … because you can reach so many new people and the presentation is so much more impactful when it's in digital video format.

I would like a lot more people to see the story because a lot of families go through the same things. So it's good it's on Facebook. A lot of people will have the chance to see it. It's a really good thing to have these stories and for people to see them.

It would be good if the stories could be played at Health Fairs or brochures or somebody to talk about things regarding the digital stories, like if people are more interested [after watching the stories] they [storytellers or people who are showing the stories] can talk to them more about how they can go about getting screening done.

## Discussion

According to our participants, digital storytelling created a way for Alaska's CHA/Ps to reflect upon and craft a personal message they wanted people within their social networks to know and understand to prevent cancer, decrease cancer risk and support community wellness. Digital stories were a comfortable and appropriate way for CHA/Ps to begin challenging conversations with family, friends and community members to bring healing to a shared cancer-related experience or to activate health promotion.

Participants indicated that digital stories offered a lens through which viewers could learn CHA/Ps’ experiences and hopes for individual and community wellness. Viewers reported digital stories as an emotionally engaging way to increase their own cancer awareness and begin conversations that often served as a springboard for reflection, insight, and cancer-prevention and risk-reduction activities. Viewers also offered constructive criticism, including their recommendation to combine the short stories with more in-depth information or medically accurate resources. Due to the short nature of digital stories, only 2–3 minutes, viewers often wanted to ask additional questions to extend their understanding.

Viewers’ comments affirmed theoretical underpinnings. The concepts of relationships and interconnectedness are central to indigenous epistemology and ontology as espoused by Wilson. Highlighted within viewers’ comments is the importance of relationships in both the CHA/P telling and sharing of their stories. Digital stories offered a way for the storyteller and the viewer to envision wellness. In addition, viewers detailed narrative characteristics as outlined within Larkey's “narrative as culture-centric health-promotion model” including realism, likeability, homophily, generation of empathy and cultural appropriateness.

## Limitations

Within the scope of this project, digital stories were developed as a tool for Alaska's CHA/Ps to share health messages within their social networks. Stories were most often viewed by family, friends and co-workers. In addition, research needs to be done to understand the power of digital stories when viewed by people who do not know the story creator.
